# Molecular Mechanisms of Cadmium Stress Resistance in Vegetable Crops

**DOI:** 10.3390/ijms26125812

**Published:** 2025-06-17

**Authors:** Mengxia Zhang, Chunjuan Dong

**Affiliations:** State Key Laboratory of Vegetable Biobreeding, Institute of Vegetables and Flowers, Chinese Academy of Agricultural Sciences, Beijing 100081, China

**Keywords:** vegetable crops, cadmium stress, molecular mechanisms, transporter genes, phytohormones, antioxidant enzymes, genetic breeding

## Abstract

Cadmium (Cd) stress poses significant threats to vegetable crops, impacting their growth, physiological processes, and safety as part of the human food chain. This review systematically summarizes the latest advances in the molecular mechanisms of vegetable crops’ resistance to Cd stress. First, physiological and biochemical responses are outlined, including growth inhibition, impaired photosynthesis, oxidative stress, disrupted nutrient absorption, altered phytohormone levels, and gene expression changes. Next, key molecular mechanisms are discussed, focusing on the roles of transporter-related genes (e.g., *NRAMP*, *HIPP*, *ABCG*), transcription factors (e.g., *HsfA1a*, *WRKY*, *ERF*), enzyme-related genes (e.g., *E3 ubiquitin ligase*, *P-type ATPase*), microRNAs (e.g., *miR398*), and potential functional genes in Cd uptake, translocation, and detoxification. Additionally, the regulatory roles of phytohormones and their analogues (e.g., brassinosteroids, gibberellin, salicylic acid) in mitigating Cd toxicity are analyzed, highlighting their involvement in antioxidant defense, gene regulation, and stress signaling pathways. Finally, future research directions are proposed, emphasizing species-specific defense mechanisms, root hair-specific Cd exclusion mechanisms, and interdisciplinary approaches integrating AI and microbiome manipulation. This review provides a comprehensive reference for enhancing Cd stress resistance in vegetable crops and promoting safe crop production.

## 1. Introduction

Cadmium (Cd) contamination of agricultural soils has emerged as a global environmental challenge, threatening crop productivity and human health through the food chain. As a non-essential heavy metal with high mobility and long soil persistence, Cd accumulates readily in vegetable crops—critical components of human diets—posing significant risks via chronic ingestion [1].

Vegetable crops respond to Cd stress through a cascade of physiological and molecular mechanisms, including growth inhibition, photosynthetic disruption, and oxidative stress. At the molecular level, studies in model plants like *Arabidopsis* and rice have identified key pathways, such as metal transporter-mediated sequestration (e.g., Natural Resistance-Associated Macrophage Protein [NRAMP], Heavy Metal ATPase [HMA]), and antioxidant enzyme activation (e.g., Superoxide Dismutase [SOD], Catalase [CAT]) [2]. However, translating these insights to specific vegetable crop groups can be challenging, as many exhibit unique genetic, metabolic, or agronomic traits that may influence their response to studied mechanisms. This variability highlights the importance of crop-specific functional validation, as the underlying molecular mechanisms—rather than being universally inapplicable—often require context-dependent verification. For instance, solanaceous crops (e.g., tomato) and crucifers (e.g., Chinese cabbage) show divergent Cd accumulation patterns, yet the underlying molecular determinants—such as tissue-specific transporter expression or hormone signaling—remain incompletely understood [3,4].

A critical gap exists in deciphering the species-specific molecular networks that govern Cd tolerance in vegetables. While transporter genes (e.g., *StNRAMP2* in potato, *Brassica chinensis HEAVY METAL-INDUCED PROTEIN 16* [*BcHIPP16*] in pak choi) and transcription factors (e.g., *HEAT SHOCK TRANSCRIPTION FACTOR A1a [HsfA1a]* in tomato) have been partially characterized [5,6], the roles of microRNAs (e.g., *Solanum lycopersicum microRNA 398* [*Sly-miR398*]), epigenetic regulators (e.g., histone demethylases), and uncharacterized genes (e.g., putative *HEAT SHOCK PROTEINS* [*HSPs*] in radish) are largely unexplored [7,8,9]. Additionally, phytohormones such as brassinosteroids (BRs) and salicylic acid (SA) play pivotal but understudied roles in integrating stress responses and modulating Cd uptake [10,11].

This review synthesizes current knowledge of Cd stress resistance in vegetable crops, focusing on physiological and biochemical responses, multi-layered molecular mechanisms (e.g., transporters, transcription factors, microRNAs), and phytohormone signaling networks. Prospective research directions, such as CRISPR-based gene editing and AI-driven modeling to enhance Cd tolerance, are discussed to inform future molecular breeding strategies. By integrating mechanistic insights with translational goals, this work aims to guide the development of sustainable solutions for Cd-stressed vegetable production.

## 2. Physiological and Biochemical Reactions of Vegetable Crops Under Cadmium Stress

### 2.1. Growth Inhibition

Cd significantly hinders the growth and development processes of vegetable crops, leading to a remarkable reduction in seed germination rate, retarded seedling growth, and a notable decrease in plant biomass. Excessive soil Cd levels impede seed sprouting, stunt root and stem elongation, and reduce both fresh and dry plant weights. Waris et al. (2023) showed that 250 µM Cd^2+^ stress led to a lower germination rate of lettuce seeds and slower growth of lettuce seedlings, as indicated by shorter root and shoot lengths, and also caused a significant decrease in the fresh and dry weights of both shoots and roots [12]. Meena et al. (2018) reported that 250 µM CdCl_2_ treatment resulted in significant reductions in the root length and volume of tomatoes [13]. Sun et al. (2024) found that 50 µM CdCl_2_ stress suppressed carrot seed germination, shortened hypocotyl length, and reduced seed viability [14]. In summary, Cd contamination imposes multifaceted constraints on vegetable crop development, disrupting seed germination, retarding growth, and reducing biomass.

### 2.2. Impaired Photosynthesis

Cd disrupts multiple aspects of photosynthesis in vegetable crops. It reduces the content of photosynthetic pigments like chlorophyll and carotenoids, hampering the light-dependent reaction. Additionally, Cd induces stomatal closure, decreasing carbon dioxide uptake, which impairs the light-independent reaction and ultimately lowers the photosynthetic rate. Hédiji et al. (2015) reported that exposure to 20 and 100 µM CdCl_2_ for 90 days led to a decrease in the chlorophyll content of tomatoes, with the higher concentration (100 µM) causing more pronounced effects [15]. Huang et al. (2022) showed that 20 µM CdCl_2_ stress significantly reduces the photosynthetic rate of pak choi seedlings, with the rate dropping to only 40% of that in control seedlings, and also causes stomatal closure and a decrease in intercellular CO_2_ concentration [16]. Shahzad et al. (2024) found that 20 mg Cd/kg soil stress disrupts photosynthesis in radish by inhibiting chlorophyll synthesis, affecting photosystem functioning, and reducing biomass production [17]. In conclusion, Cd disrupts plant photosynthesis through multiple pathways, severely impairing the photosynthetic physiological processes and growth of vegetable crops.

### 2.3. Oxidative Stress Response and Altered Enzyme Activity

Under Cd stress, vegetable crops overproduce reactive oxygen species (ROS) such as hydrogen peroxide (H_2_O_2_), superoxide anion (O_2_^−^), and hydroxyl radical (·OH). This triggers oxidative stress, exacerbates lipid peroxidation, increases the level of malondialdehyde (MDA), and damages the structure and function of cell membranes. Meanwhile, Cd binds to the active sites of antioxidant enzymes like SOD, peroxidase (POD), and CAT, disrupting the antioxidant enzyme system. To cope with excessive ROS, the activities of these enzymes in different vegetable crops may either increase or decrease. For example, Dong et al. (2006) found that Cd stress at 1–10 µM induced the accumulation of MDA and an increase in antioxidant enzyme activities in tomato seedlings [18]. Cui et al. (2023) reported that Cd stress at 6.24 mg/kg soil increased the contents of H_2_O_2_, MDA, and O_2_^−^ in pak choi, causing oxidative damage and affecting the activities of POD, SOD, and CAT, with the enzymes generally increasing their activities to reduce oxidative damage [19]. Huang et al. (2022) [16] showed that Cd stress at 20 mg/kg soil in radish led to increased oxidative stress, as indicated by elevated levels of H_2_O_2_ and MDA, and also altered the activities of SOD, POD, and CAT [17]. In conclusion, Cd stress in vegetable crops disrupts the balance between ROS generation and antioxidant defense, leading to oxidative damage and diverse responses in antioxidant enzyme activities, which vary among different vegetable species.

### 2.4. Disordered Nutrient Element Absorption

Cd competes with nutrient elements such as calcium (Ca), magnesium (Mg), iron (Fe), and zinc (Zn) in vegetable crops for absorption sites or transport proteins, thereby disrupting the normal absorption and transport processes and causing nutrient imbalance. For instance, Cd can interfere with Fe absorption in certain vegetable crops, inducing Fe-deficiency chlorosis and inhibiting photosynthesis. Research has provided evidence for these effects: Hédiji et al. (2015) documented the changes in the contents of Ca, potassium (K), and Mg in tomato plants under varying Cd exposures, including 50 and 100 µM CdCl_2_ [15]; Ma et al. (2025) reported that in radishes, excessive Cd absorption at 50 µM CdCl_2_ disrupts the normal uptake of elements like Fe and Manganese (Mn), ultimately hindering the growth and development [20]; and Wu et al. (2021) found that 50 µM CdCl_2_ stress affects nutrient element absorption in pak choi, increasing Fe concentration, reducing Zn concentration, and altering Mn absorption [21]. In summary, Cd’s interference with the absorption and transport of nutrient elements through competitive mechanisms results in diverse and vegetable–crop-specific nutrient imbalances, significantly impacting the physiological functions and growth of vegetable crops.

### 2.5. Changes in Phytohormone Levels

Plant hormones are essential for vegetable crops to respond to Cd stress. Cd impacts the synthesis, metabolism, and signal transduction of various hormones, including auxin (IAA), gibberellin (GA), cytokinin (CK), abscisic acid (ABA), SA, BR, jasmonic acid (JA), and ethylene (ET), thereby regulating the growth and defense mechanisms of vegetable crops. Research has shown diverse effects of Cd on hormone levels across different vegetable species. For instance, Asaf et al. (2023) discovered that 1 mM and 2 mM Cd stress increased the levels of stress-related phytohormones like JA, ABA, and ET in tomato plants, and this increase could be mitigated by inoculating the endophytic fungus SL1 [22]. In contrast, Yang et al. (2024) reported that 250 μM Cd stress decreased the JA content in potato plants, while the content of BR increased, and JA content was found to have positive correlations with IAA and SA, and negative correlations with BR, ET, ABA, GA, etc. [23]. Moreover, Tang et al. (2023) demonstrated that under 50 mmol/L Cd stress, lettuce exhibited increased expression of *SALICYLIC ACID METHYLTRANSFERASE* (*SAMT*) involved in SA synthesis and decreased expression of *WRKY DNA-BINDING PROTEIN 6* (*WRKY6*) related to plant hormone-mediated regulation, thereby disrupting normal hormone-associated physiological functions under stress [11]. In summary, Cd stress exerts complex and varied impacts on the hormones of vegetable crops, influencing different aspects of their physiological processes, and understanding these relationships is crucial for developing effective strategies to improve the Cd tolerance of vegetable crops.

### 2.6. Changes in Gene Expression Regulation

Cd stress triggers diverse gene expression changes in vegetable crops, involving genes related to metal detoxification such as those encoding metallothioneins and phytochelatins, antioxidant enzyme genes, transporter genes, and genes related to photosynthesis, cell cycle, and signal transduction, with these transcriptional adjustments enabling vegetable crops to adapt to Cd stress or mitigate its toxicity. For example, Meena et al. (2018) found that Cd stress upregulates the *LeNRAMP3* gene in tomato with peak expression at 250 μM Cd, indicating its role in Cd translocation from roots to leaves [13]. Kim et al. (2007) demonstrated that 40 μM Cd stress induces the *Brassica rapa type-1 metallothionein* gene (*BrMT1*), particularly in the roots of Chinese cabbage, and enhances Cd resistance in transgenic yeast and *Arabidopsis* [24]. Sun et al. (2024) showed that 50 μM Cd stress upregulates the *Daucus carota V-MYB AVIAN MYELOBLASTOSIS VIRAL ONCOGENE HOMOLOG 62* (*DcMYB62*) gene in carrot, promoting carotenoid biosynthesis and enhancing Cd tolerance by activating genes related to ABA, hydrogen sulfide (H_2_S) production, and heavy metal resistance (e.g., *Arabidopsis thaliana NICOTIANAMINE SYNTHASE 4* [*AtNAS4*]) in transgenic *Arabidopsis* [25]. These studies collectively illustrate how gene expression reprogramming underlies the adaptive strategies of vegetable crops against Cd stress, with diverse genes contributing to detoxification, transport, and stress tolerance through interconnected molecular pathways.

**Figure 1:**
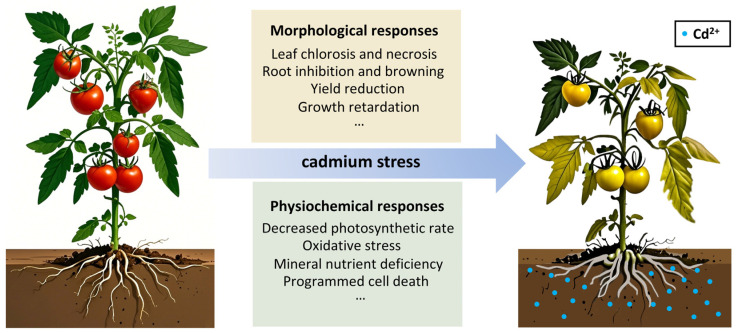
Responses of tomato plants to cadmium stress: morphological and physiochemical changes.

Under Cd stress, vegetable crops exhibit diverse physiological/biochemical responses across species/varieties, as illustrated by tomato plants’ morphological and physicochemical changes (see Figure 1). Deciphering these differences requires exploring multi-gene regulatory networks governing Cd tolerance, including transporter genes, signaling pathways, antioxidant systems, etc. Such insights underpin strategies to enhance plant resilience and reduce Cd accumulation in vegetables.

## 3. Molecular Mechanisms of Multiple Genes in Regulating Vegetable Crops’ Response to Cadmium Stress

### 3.1. Transporter-Related Genes

Cd stress poses significant challenges to vegetable crops, with transporter-related genes playing pivotal roles in regulating Cd uptake, translocation, and tolerance. In potato, the *StNRAMP2* gene (*NRAMP*s family) influences Cd accumulation in a tissue-specific manner: silencing *StNRAMP2* increases Cd in tubers but decreases it in other tissues under 100 mg/kg Cd stress, while heterologous overexpression in tomato enhances Cd content under 100 mg/kg Cd stress, confirming its role in metal ion transport and redistribution [5]. In pak choi, the plasma membrane-localized BcHIPP16 (HIPP family) acts as a metal chaperone to promote Cd and copper (Cu) uptake. Transgenic *Arabidopsis* expressing *BcHIPP16* shows enhanced Cd^2+^ influx and accumulation under 1 μM or 5 μM Cd treatment, linking its function to direct metal ion translocation across membranes [6]. In radish, the ATP-Binding Cassette Subfamily G (ABCG) subfamily member *Raphanus sativus* PLEIOTROPIC DRUG RESISTANCE 8 (RsPDR8) functions as a Cd efflux pump. Overexpression in *Arabidopsis* reduces Cd accumulation in roots and shoots, improves root elongation under 50 μM or 100 μM Cd stress, and enhances ROS scavenging via upregulated antioxidant enzymes (e.g., Ascorbate Peroxidase [APX], SOD), highlighting its dual role in metal extrusion and stress tolerance [26]. These findings collectively highlight the multifaceted roles of transporter-related genes in modulating Cd stress responses, encompassing tissue-specific metal allocation, membrane transport dynamics, and antioxidant defense coordination. For a detailed summary of these genes and their specific functions in vegetable crops under Cd stress, refer to Table 1.

### 3.2. Transcription Factors

Cd stress represents a major threat to vegetable crops, with transcription factors (TFs) emerging as critical regulators of gene expression to mitigate Cd toxicity. In tomato, the heat shock transcription factor HsfA1a directly binds to the promoter of *CAFFEIC ACID O-METHYLTRANSFERASE 1* (*COMT1*) via heat shock elements (HSEs) to upregulate melatonin biosynthesis, which in turn enhances Cd tolerance by promoting phytochelatin biosynthesis, vacuolar Cd sequestration, and activating HSPs such as HSP20 and HSP70 under 100 μM Cd^2+^ treatment for 15 days [28]. In pepper, CaWRKY41 forms a positive feedback loop with H_2_O_2_: Cd stress (e.g., 25 μM CdSO_4_) induces *CaWRKY41* expression, which upregulates NADPH oxidase genes (*RESPIRATORY BURST OXIDASE HOMOLOG C/F* [*RBOHC/F*]) to enhance H_2_O_2_ accumulation, while H_2_O_2_ further activates *CaWRKY41*. This loop improves resistance to *Ralstonia solanacearum* but increases Cd sensitivity by upregulating Zn transporters (*ZRT/IRT-like Protein 3/4/9* [*ZIP3/4/9*]), leading to enhanced Cd uptake [29]. In bean, the ethylene response factor *Phaseolus vulgaris* ETHYLENE RESPONSE FACTOR 15 (PvERF15) binds to an AC-rich element in the *Phaseolus vulgaris METAL RESPONSE ELEMENT-BINDING TRANSCRIPTION FACTOR* 1 (*PvMTF-1*) promoter to activate its expression for enhanced Cd tolerance, while PvMTF-1 promotes tryptophan (Trp) biosynthesis and Cd detoxification, and RNAi-mediated *PvERF15* knockdown reduces *PvMTF-1* expression and Cd tolerance in transient assays with 200 μM CdCl_2_ treatment [30]. These studies illustrate that TFs orchestrate multifaceted responses to Cd stress, integrating hormone signaling (e.g., ethylene), ROS homeostasis, and metal transporter regulation. For a comprehensive overview of transcription factors and their specific regulatory roles in vegetable crops under Cd stress, including additional genes and mechanisms, refer to Table 2.

### 3.3. Enzyme-Encoding Genes

Cd stress poses substantial threats to vegetable crops, and enzyme-encoding genes play crucial roles in regulating Cd tolerance, detoxification, and metabolic adaptation. In tomato, the RING E3 ubiquitin ligase gene *SlRING1* confers Cd tolerance by reducing Cd accumulation and oxidative stress through decreasing root/shoot Cd content, enhancing antioxidant enzyme activities, and improving photosynthetic efficiency under 100 μM CdCl_2_ stress, with its E3 ligase activity and subcellular localization (plasma membrane and nucleus) indicating roles in protein ubiquitination and stress signaling [36,37]. In pepper, the Golgi/ER-localized heavy metal-transporting ATPase CaHMA1 (P-type ATPase family) promotes fruit Cd accumulation via histidine/glutamic acid residues in its HEGGILLVC motif under 5 mg/kg soil Cd, as VIGS-mediated silencing reduces fruit Cd content while *Arabidopsis* overexpression increases Cd uptake, confirming its role in metal ion translocation [38]. In Chinese flowering cabbage, the peptidemethionine sulfoxide reductase BpPMSR3 enhances Cd tolerance by reducing methionine sulfoxide (MetSO) and increasing glutathione/phytochelatin levels under 50 μM CdCl_2_, with transgenic *Arabidopsis* overexpressing *BpPMSR3* exhibiting improved root growth and upregulated Cd-tolerance genes (*AtHMA3*, *AtNramp1*), indicating roles in oxidative stress mitigation and metal detoxification [39]. These studies illustrate the diverse functions of enzyme-related genes in Cd stress responses, including oxidative stress regulation, protein modification, and metal ion transport. For a comprehensive overview of these genes and their regulatory roles in vegetable crops under Cd stress, refer to Table 3.

### 3.4. MicroRNAs and Other Types of Genes

Cd stress presents substantial challenges to vegetable crops, where microRNAs and various genes play crucial roles in governing Cd uptake, translocation, and tolerance. In tomato, 100 µM Cd stress downregulates *Sly-miR398* to upregulate *COPPER/ZINC SUPEROXIDE DISMUTASE 1* (*CSD1*) and *SOD*, while transgenic overexpression of *Sly-miR398* promotes Cd uptake (via *Iron-Regulated Transporter 1/2 [IRT1/2]* and *NRAMP2*) and reduces vacuolar sequestration (via *HMA3*), leading to elevated shoot Cd and oxidative damage [7]. In water spinach, a 5 mg/L Cd treatment leads to the downregulation of *Ipomoea aquatica microRNA 4-3p* (*IamiR-4-3p*), which in turn suppresses *GLUTATHIONE S-TRANSFERASE 3* (*GST3*) and *ABA-INDUCED WHEAT PLASMA MEMBRANE POLYPEPTIDE-19* (*AWPM19*)*-like*. This results in higher Cd accumulation in transgenic *Arabidopsis* through weakened apoplastic barriers and increased oxidative stress, thereby enhancing metal translocation [47]. In Chinese cabbage, *Brassica rapa STRESS-SEVENTY SUBFAMILY A 4c* (*BrSSA4c*) (an HSP70 family gene) enhances Cd tolerance likely through altered metal transport or stress signaling, distinct from its mechanism in yeast where ScSSA4 translocates to the nucleus to interact with PORE MEMBRANE PROTEIN 34 (POM34) and activate Vacuolar H+-ATPase Subunit S1 (VHS1) for reduced Cd accumulation under 50 µM Cd [4]. These studies highlight the diverse roles of microRNAs and genes in Cd stress responses, including transcriptional regulation of transporters, redox balance modulation, and subcellular metal compartmentalization. For a detailed summary of these genes and their specific functions in vegetable crops under Cd stress, refer to Table 4.

### 3.5. Putative Functional Genes

While significant progress has been made in identifying well-characterized genes involved in Cd stress responses in vegetable crops, a growing body of research, including gene family analyses, comparative genomics, and targeted transcriptomic studies, has identified numerous genes with potential functions in Cd stress responses that remain to be fully elucidated. These genes were detected through transcriptomic and proteomic profiling under Cd stress treatments, often showing differential expression patterns indicative of stress responses. However, due to limited functional validation experiments, their specific roles in Cd uptake, translocation, sequestration, or detoxification remain speculative. For instance, certain members of the NRAMP gene family demonstrated altered expression in multiple studies but lacked transgenic or knockout confirmation [3,50,51]. Despite this, these genes represent promising targets for future research to dissect the complex molecular mechanisms underlying vegetable crops’ tolerance to Cd stress. Their identification not only expands our understanding of plant stress biology but also holds potential for developing biotechnological strategies to enhance crop safety and quality. A comprehensive list of these putative functional genes, summarized from recent literature, is presented in Table 5 for further reference.

## 4. Molecular Mechanisms of Phytohormones and Their Analogues in Regulating Vegetable Crops’ Response to Cadmium Stress

Phytohormones serve as pivotal regulators in orchestrating plant adaptive responses to Cd stress, integrating physiological adjustments and molecular mechanisms across diverse vegetable species. For example, BRs such as 24-epibrassinolide (24-EBL) and 3-epibrassinolide (3-EBL) exhibit multifaceted roles in cucumber. 24-EBL mitigates Cd toxicity by enhancing antioxidant enzyme activities (e.g., SOD, POD) and modulating gene expression related to ethylene and auxin biosynthesis, thereby reducing lipid peroxidation and Cd uptake [92]. Similarly, 3-EBL improves photosynthetic efficiency and water relations while regulating stress-responsive genes, linking hormonal signaling to physiological resilience [93]. In lettuce, GA and SA adopt distinct strategies: GA downregulates metal transporter genes (*IRT1*, *Nramp1*, *HMA2*, *HMA4*) to restrict Cd uptake and translocation [94], whereas SA enhances photosynthesis, antioxidant defenses, and osmolyte accumulation while suppressing *Nramp5* and *HMA4* expression to limit Cd accumulation [11]. These mechanisms, summarized in Table 6, highlight the versatility of phytohormones in fine-tuning stress tolerance across vegetable crops.

**Figure 2:**
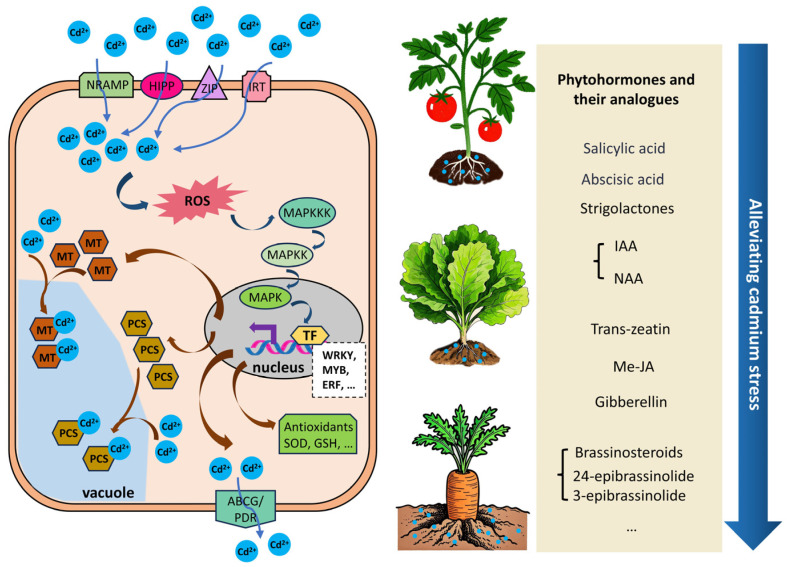
Cadmium-induced signal pathways and phytohormone-mediated alleviation in vegetable crops.

In preceding sections, the molecular mechanisms of stress-responsive genes (e.g., *Nramp*, *HMA*, antioxidant enzyme genes) and the regulatory roles of phytohormones (e.g., BRs, GA, SA) in vegetable crops’ Cd stress tolerance were systematically discussed. These two thematic areas—gene-driven stress responses and hormone-mediated alleviation strategies—are concisely summarized in Figure 2.

## 5. Conclusions and Future Directions

This review systematically summarizes current knowledge of vegetable crops’ responses to Cd stress, covering their physiological and biochemical reactions, multigenic regulatory mechanisms, and phytohormone-mediated signaling pathways. Building on these foundations, future research on the molecular mechanisms of cadmium resistance in vegetable crops should address several interconnected scientific challenges with targeted methodologies.

A pressing challenge lies in decoding the species-specific defense strategies of vegetable crops. Despite diverse Cd tolerance profiles, the molecular basis of this diversity remains unclear. Integrating transcriptomics, proteomics, and metabolomics with CRISPR-Cas9 could dissect crop-specific defense modules. For example, in tomato, CRISPR-mediated knockout of root-localized HMA transporters, such as SlHMA2, could clarify their role in Cd sequestration. Promoter analysis might reveal cis-elements co-regulated with detoxification genes, like *PHYTOCHELATIN SYNTHASE* (*PCS*). These insights could be translated to other vegetables, such as lettuce and radish, using optimized Agrobacterium-mediated transformation protocols [102,103], thus establishing a “gene toolkit” for Cd-resistant breeding.

Another critical area for investigation is the root hair-specific adaptation mechanisms for Cd exclusion. Vegetable roots rely on root hairs for nutrient uptake, yet how these structures modulate Cd influx remains uncharacterized. Advanced live-cell imaging techniques, such as confocal laser scanning microscopy, can be applied to track dynamic changes in ZIP transporter localization, antioxidant enzyme activity in root hair tip cells, and cell wall modifications that trap Cd ions at the apoplastic barrier. These studies would resolve whether root hairs actively “block” Cd uptake or serve as passive diffusion barriers, providing a basis for engineering “Cd-reflective” root architectures.

Engineering transdisciplinary solutions via AI and microbiome manipulation also holds great promise. Machine learning models trained on multi-omics datasets could predict optimal root exudate compositions to suppress Cd solubility. Meanwhile, AI-driven protein design could optimize phytochelatin sequences for enhanced Cd-binding affinity, overcoming natural detoxification bottlenecks. Identifying rhizobacteria, such as *Pseudomonas* spp., that secrete Cd-chelating compounds or induce systemic resistance via salicylic acid signaling is crucial. Metagenomic analyses can map microbial genes involved in Cd methylation/oxidation, enabling co-cultivation strategies that convert bioavailable Cd into stable mineral forms. Future investigations could also explore the synergistic integration of soil amendment strategies (e.g., biochar-mediated Cd immobilization [17] or microbial community modulation [22]) with plant molecular mechanisms, such as engineering metal transporter genes (e.g., *StNRAMP2* [5]) or phytohormone signaling pathways (e.g., BRs [10]), to develop comprehensive Cd tolerance strategies.

By linking gene regulatory networks, such as metal transporter hierarchies, to AI-optimized rhizosphere engineering, this framework enables precision breeding for “Cd-resilient” vegetables. Epigenetic studies, like bisulfite sequencing, could identify Cd-responsive DNA methylation hotspots in spinach, facilitating marker-assisted selection for stress “memory” traits. The development of smart biosensors, combining soil Cd nanosensors and plant-derived stress biomarkers, such as Cd-bound phytochelatins in xylem sap, would enable real-time irrigation and nutrient management. This integrative approach balances fundamental discovery, such as the molecular basis of Zn/Cd discrimination in roots, with translational innovation, offering dual benefits: reducing dietary Cd exposure through crop biofortification while preserving each species’ unique adaptive strategies. Success will ultimately depend on fostering collaborations between molecular biologists, computational ecologists, and agricultural engineers to bridge lab-scale mechanistic insights with field-applicable solutions.

## Figures and Tables

**Table 1:** Regulatory roles of transporter-related genes in vegetable crops under cadmium stress.

Vegetable Name	Gene	Tissue Specificity ^a^	Localization ^b^	Core Mechanism Summary ^f^	Key Functional Impact	Ref.
Tomato	*LeNRAMP3*	-	PM	Interacts with NAS/FRO/IRT1 complex; mediates Fe/Cd transport and signaling	↑ heavy metal translocation	[13]
Potato	*StNRAMP2*	-	-	Regulates Cd partitioning: silencing ↑ Cd in tubers, overexpression ↑ Cd in shoots	Modulates Cd accumulation patterns	[5]
Pak choi	*BcHIPP16*	Whole seedling	PM	Direct Cd^2+^ influx promoter in roots	↑ Cd uptake efficiency	[6]
Chinese cabbage	*BcNRAMP1*	Whole plant	PM	Activated by Cd/Mn deficiency; ↑ Cd/Mn root uptake	Dual regulation under metal stress	[27]
Chinese cabbage	*BrMT1*	Roots/flowers ^c^	Cyto/Chl ^d^	Cysteine-rich Cd chelation; chloroplast targeting ↑ ROS scavenging	Confers Cd resistance via subcellular targeting	[24]
Radish	*RsPDR8*	Vascular tissues ^e^	PM	↑ Cd efflux via PM transport; ↑ ROS scavenging & membrane stability	↓ cellular Cd accumulation	[26]
Radish	*RsNRAMP5*	Vascular cambium	PM	↑ Cd influx; differential regulation of ROS/proline genes	Mediates Cd uptake-toxicity balance	[20]

^a^ Tissue expression sites abbreviated if absent (“-”) or non-specific (“whole plant”). ^b^ PM: Plasma membrane; Cyto: Cytosol; Chl: Chloroplast. ^c^ High in roots/flowers, low in old leaves/stems. ^d^ Localization dependent on expression system (Arabidopsis). ^e^ Xylem/phloem/cambium. ^f^ Arrows (↑/↓) denote enhancement/reduction of processes.

**Table 2:** Regulatory roles of transcription factors in vegetable crops under cadmium stress.

Vegetable Name	Gene	Tissue Specificity ^a^	Localization ^b^	Core Mechanism Summary ^c^	Key Functional Impact	Ref.
Tomato	*LeSPL-CNR*	-	-	↓ Cd acquisition via NO-mediated repression of nitrate reductase	↓ Cd accumulation (via iron-uptake suppression)	[31]
Tomato	*HsfA1a*	-	-	↑ Melatonin biosynthesis (COMT1) and HSP expression under Cd stress	↑ Cd tolerance (via upregulating HSP)	[28]
Potato	*StWRKY6*	-	Nu	↑ Antioxidant enzymes and photosynthesis regulation for stress resilience	↑ Cd tolerance; ↓ Cd accumulation	[32]
Pepper	*CaPF1*	-	-	↑ Antioxidant enzyme activity and ↓ lipid peroxidation under Cd stress	↑ Cd tolerance in transgenic plants	[33]
Pepper	*CaWRKY41*	Roots, leaves, (shoots, flowers)	Nu (*Arabidopsis*)	↑ H_2_O_2_ accumulation and ↑ Zn transporters activation for Cd uptake	↓ Cd tolerance; ↑ Cd uptake	[29]
Carrot	*DcMYB62*	-	Nu	↑ Carotenoid/ABA/H_2_S biosynthesis, ↓ ROS, and ↑ stomatal closure activation	↓ ROS; ↑ expression of heavy metal resistance genes	[25]
Bean	*PvERF104*	-	Nu	↓ Cd-induced lipid peroxidation and ↑ regulation of MRE-containing Cd response genes	↓ Cd accumulation; ↑ stress tolerance	[34]
Bean	*PvMTF-1*	-	Nu	↑ Tryptophan synthesis via *ASA2*-mediated pathway by binding *ASA2* promoter under Cd stress	↓ Cd accumulation	[35]
Bean	*PvERF15*	-	Nu	↑ *PvMTF-1* promoter activation via ACE-binding for Cd stress response regulation	Forms transcriptional pathway for Cd response	[30]

^a^ Tissue expression sites abbreviated if absent (“-”). ^b^ Nu: Nucleus; “*Arabidopsis*” denotes experimental system. ^c^ Arrows (↑/↓) denote enhancement/reduction of processes.

**Table 3:** Regulatory roles of enzyme-encoding genes in vegetable crops under cadmium stress.

Vegetable Name	Gene	Tissue Specificity ^a^	Localization ^b^	Core Mechanism Summary ^d^	Key Functional Impact	Ref.
Tomato	*SlRING1*	-	PM/Nu	E3 ligase-mediated ubiquitination ↑ antioxidant/detoxification pathways	↓ Cd accumulation & oxidative stress	[36,37]
Tomato	*Sl1*	Roots ^c^	PM	E3 ligase-mediated suppression of metal transporters ↑ antioxidant activity	↓ Root Cd uptake	[40]
Tomato	*Tgase*	Leaves/flowers	Mt/CW/Chl	↑ Polyamine/NO accumulation & cell wall modification	↑ Cd chelation capacity	[41,42]
Tomato	*SlSPR2*	-	-	JA-dependent regulation ↑ Cd-responsive metabolic pathways	Maintains physiological homeostasis	[43]
Tomato	*SlMAPK3*	Stems/roots	-	MAPK signaling ↑ antioxidant activation & root morphology regulation	↑ Root function under stress	[44]
Tomato	*SlJMJ524*	Leaves/flowers	-	Epigenetic regulation ↓ metal transporters & ↑ GSH-PC synthesis	↑ Cd sequestration	[45]
Tomato	*SlJMJ18/23*	Flowers (*SlJMJ18*) ^c^, young leaves (*SlJMJ23*) ^c^	-	Epigenetic regulation ↓ metal transporters (ZIP1, IRT1) & ↑ antioxidant/phenol synthesis	↑ Antioxidant capacity & phenol synthesis	[8]
Pepper	*CaHMA1*	-	GA/ER	Heavy metal-binding domain ↑ Cd accumulation	↑ Cd translocation to fruits	[38]
Chinese cabbage	*BpPMSR3*	-	-	Methionine redox regulation ↑ GSH synthesis	↑ Cd detoxification	[39]
Onion	*AcGCL*	Roots ^c^	-	↑ GSH/PC synthesis & protection of FOS metabolism	Maintains carbohydrate metabolism	[46]

^a^ Tissue expression sites abbreviated if absent (“-”). ^b^ PM: Plasma membrane; Nu: Nucleus; Mt: Mitochondria; CW: Cell wall; Chl: Chloroplast; GA: Golgi apparatus; ER: Endoplasmic reticulum. ^c^ Highest expression compared to other tissues. ^d^ Arrows (↑/↓) denote enhancement/reduction of processes.

**Table 4:** Regulatory roles of microRNAs and other types of genes in vegetable crops under cadmium stress.

Vegetable Name	Gene	Tissue Specificity ^a^	Localization ^b^	Core Mechanism Summary ^c^	Key Functional Impact	Ref.
Tomato	*Sly-miR398*	Roots, stems	-	↓ regulated under Cd stress; ↑ *CSD1*/*SOD* expression to ↑ antioxidant defense; ↓ Cd uptake/translocation genes.	↓ Oxidative damage; ↑ growth recovery	[7]
Water spinach	*IamiR-4-3p*	-	-	↓ regulates *GST3* and *AWPM19*-like, causing ↑ oxidative damage and ↑ Cd uptake/translocation.	↑ Cd toxicity; ↓ apoplastic barrier	[47]
Tomato	*SlTCMP-1*	Flower buds, leaves, fruits	-	Induced by Cd stress; interacts with HIPP26 to ↑ ROS scavenging genes and ↓ Cd translocation.	↑ Cd stress response	[48]
Tomato	*SlSGR2*	Various tissues	Chloroplast	Inhibits chlorophyll degradation; ↓ MDA content; ↑ antioxidant enzyme activity under Cd stress.	↑ Cd tolerance; ↓ chlorophyll loss	[49]
Chinese cabbage	*BrSSA4c*	-	-	Overexpression ↑ Cd tolerance via activation of cis elements.	↑ Cd resilience	[4]

^a^ Tissue expression sites abbreviated if absent (“-”). ^b^ PM: Plasma membrane; “-” indicates unavailable localization data. ^c^ Arrows (↑/↓) denote enhancement/reduction of processes.

**Table 5:** Putative functional genes in vegetable crops under cadmium stress.

Vegetable Name	Botanical Name	Genes	Ref(s).
bean	*Phaseolus vulgaris* L.	*PvSR3*; *PvSR2*	[52,53]
Chinese cabbage	*Brassica rapa* L.	*BrAHL24*; *BrHO1*; *BrMYB116*	[54,55,56]
cucumber	*Cucumis sativus* L.	*DAO*; *CsNramp1*, *CsNramp4*, *CsZIP1*, *CsZIP8*, *CsHMA5*, *CsHMA2*, *CsHMA7*; *CsMT4*	[50,57,58]
flowering Chinese cabbage	*Brassica rapa* L. *Chinensis*	*BrMT*s, *BrPCS*s	[59]
garlic	*Allium sativum* L.	*AsPCS1*, *AsMT2a*; *AsMT2b*	[60,61]
kale	*Brassica oleracea* var. *acephala*	*OPT3*, *YSL3*	[62]
lettuce	*Lactuca sativa* L.	*LsXTH6*, *LsXTH7*, *LsXTH8*, *LsXTH32*, and *LsXTH33*; *LsAPX*s, *LsSOD*s; *LsZIP1*, *LsZIP3*, *LsZIP10*, *LsZIP12*, *LsZIP13*, *LsZIP17*, *LsZIP19*	[63,64,65]
melon	*Cucumis melo* L.	*CmMlo1*	[66]
mung bean	*Vigna radiata* (L.) R. Wilczek	*VrCOX*s	[67]
pak choi	*Brassica chinensis* L.	*Fe SOD1*, *POD A2/44/54/62*, and *GST1*; *BcIRT1*, *BcZIP2*; *BcGSTU*s	[21,68,69]
pepper	*Capsicum annuum* L.	*CaNRAMP5*, *CaCOMT1*	[51]
potato	*Solanum tuberosum* L.	*StCAD*s; *StOPR1*, *StJAZ14*; *StAP2/ERF* genes; *StNRAMP*s; *StSRO*s *5/6*; *StABC*s; *StDREB1*, *StDREB2*	[23,70,71,72,73,74,75,76,77]
radish	*Raphanus sativus* L.	*RsHSP70-5*, *RsHSP70-14*, *RsHSP70-21*, *RsHSP70-32*; *RsMATE37-a*, *RsMATE21*, *RsMATE43-c*, *RsMATE49-b*, *RsMATE31-b*, *RsMATE33*, *RsMATE46-c*, *RsMATE13-a*, *RsMATE16-b*, *RsMATE43-a*, *RsMATE27*, *RsMATE35-a*, *RsMATE40-b*, *RsMATE13-c* and *RsMATE26*; *RsZIP* genes; *WRKY6*, *WRKY28-like*, *WRKY33*, *MYB16*, *bHLH143*, *ERF—rap2.7*, *PIN1*, *MRP*, *ABC* transporter genes, *GST* and *LCC4*	[9,78,79,80]
tomato	*Solanum lycopersicum* L.	*Solyc05g051550, Solyc02g077370, Solyc04g009440, Solyc12g099130, Solyc04g077960, Solyc04g051690, Solyc08g078180, Solyc07g045030, Solyc05g015850, Solyc05g053330, Solyc12g013640, Solyc07g065320, Solyc11g012700, Solyc11g069735, Solyc01g104820*; *COMT*, *PCS*; *SlDML1, SlDML2, SlDML3, SlDML4, SlMET1, SlDRM1L, SlDRM5 and SlDRM1L1; SlNRAMP1-5; SlMT1-4; SlWRKY76, SlWRKY38, SlWRKY46, SlWRKY19, SlWRKY33, SlWRKY35, SlWRKY45, SlWRKY51, SlWRKY55, SlbHLH*s; *miR166a, miR395b*; *FW2.2/CELL NUMBER REGULATOR (CNR)*; *SlHIPP7/21/26/32*; *SlERF1*	[3,81,82,83,84,85,86,87,88,89]
turnip	*Brassica rapa* L.	*BrHMA*s	[90]
water spinach	*Ipomoea aquatica* Forsk.	*IaMT2*, *IaMT3*	[91]

**Table 6:** Regulatory roles of phytohormones and their analogues in vegetable crops under cadmium stress.

Vegetable Name	Botanical Name	Substance Name	Core Mechanism Summary ^a^	Key Functional Impact	Ref(s).
cherry tomato	*Solanum lycopersicum* var. *cerasiforme*	Melatonin and brassinosteroids (BRs)	↓ Cd content in shoots; ↑ antioxidant enzyme activities/gene expression; regulates K+ balance	↑ Cd detoxification	[10]
cucumber	*Cucumis sativus* L.	24-epibrassinolide (24-EBL)	↑ Antioxidant system; regulates ethylene/IAA biosynthesis genes; ↓ lipid peroxidation and Cd uptake	↓ Cd toxicity	[92]
cucumber	*Cucumis sativus* L.	3-epibrassinolide (3-EBL)	↑ Antioxidant enzymes; regulates ethylene/auxin biosynthesis genes; improves photosynthesis/water physiology	↑ Cd stress resilience	[93]
cucumber	*Cucumis sativus* L.	Me-JA and H_2_O_2_	↑ Cell cycle-related gene expression; activates adventitious rooting through H_2_O_2_ signaling (inhibited by CAT/DPI)	↑ Root development	[95]
lettuce	*Lactuca sativa* L.	Gibberellin (GA)	↓ IRT1/Nramp1 expression (↓ Cd uptake); ↓ HMA2/HMA4 expression (↓ root-to-shoot translocation)	↑ Cd tolerance	[94]
lettuce	*Lactuca sativa* L.	salicylic acid (SA)	Regulates Nramp5/HMA4/SAMT expression; ↑ photosynthesis; modulates antioxidant/osmotic systems	↓ Cd accumulation; ↑ oxidative defense	[11]
melon	*Cucumis melo* L.	SLs	↓ Cd stress in roots by regulating redox-related genes (POD, LOX), transcription factors (MYB, AP2/ERF), and ↑ JA biosynthesis/flavonoid pathways	↑ Antioxidant capacity; ↓ oxidative damage	[96]
mung bean	*Vigna radiata* L.	abscisic acid (ABA)	↑ Antioxidative enzymes/IAA oxidase activity; regulates cell wall/secondary metabolism genes	↑ Adventitious root formation	[97]
mung bean	*Vigna radiata* L.	SA	↑ SOD/POD/CAT/APX activities; modulates phytohormones (↑ ABA/JA, ↓ ethylene); regulates osmolyte metabolism	↑ Physiological homeostasis	[98]
pepper	*Capsicum annuum* L.	Trans-zeatin (tr-Z) and silymarin (Sm)	↓ Cd uptake/accumulation; ↑ antioxidant enzymes/compounds; modulates stress-responsive gene expression	↑ Antioxidant defense system	[99]
radish	*Raphanus sativus* L.	Strigolactone (SL) and acidified biochar (AB)	SL regulates root architecture and chloroplast development; AB adsorbs Cd ions and improves soil conditions; combination ↑ antioxidant activities	↑ Cd stress tolerance	[17]
tomato	*Solanum lycopersicum* L.	IAA	↑ NO accumulation → activates AsA-GSH cycle; ↓ Cd accumulation/oxidative markers	↑ Photosynthetic protection	[100]
tomato	*Solanum lycopersicum* L.	α-naphthaleneacetic acid (NAA)	Regulates defense genes in shoots and oxidoreductase/auxin-response genes in roots; modulates antioxidant system/Cd transport	↓ Cd-induced damage	[101]

^a^ Arrows (↑/↓) denote enhancement/reduction of processes.
